# Pioglitazone Increases Whole Body Insulin Sensitivity in Obese, Insulin-Resistant Rhesus Monkeys

**DOI:** 10.1371/journal.pone.0126642

**Published:** 2015-05-08

**Authors:** Effie Tozzo, Gowri Bhat, Kyeongmi Cheon, Raul C. Camacho

**Affiliations:** 1 Department of Diabetes, Merck Research Laboratories, Kenilworth, New Jersey, United States of America; 2 Department of Molecular Biomarkers, Merck Research Laboratories, Kenilworth, New Jersey, United States of America; 3 Department of Biometrics Research, Merck Research Laboratories, West Point, Pennsylvania, United States of America; Kobe University, JAPAN

## Abstract

Hyperinsulinemic-euglycemic clamps are considered the "gold standard" for assessing whole body insulin sensitivity. When used in combination with tracer dilution techniques and physiological insulin concentrations, insulin sensitization can be dissected and attributed to hepatic and peripheral (primarily muscle) effects. Non-human primates (NHPs), such as rhesus monkeys, are the closest pre-clinical species to humans, and thus serve as an ideal model for testing of compound efficacy to support translation to human efficacy. We determined insulin infusion rates that resulted in high physiological insulin concentrations that elicited maximal pharmacodynamic responses during hyperinsulinemic-euglycemic clamps. These rates were then used with [U-^13^C]-D-glucose, to assess and document the degrees of hepatic and peripheral insulin resistance between healthy and insulin-resistant, dysmetabolic NHPs. Next, dysmetabolic NHPs were treated for 28 days with pioglitazone (3 mg/kg) and again had their insulin sensitivity assessed, illustrating a significant improvement in hepatic and peripheral insulin sensitivity. This coincided with a significant increase in insulin clearance, and normalization of circulating adiponectin. In conclusion, we have determined a physiological clamp paradigm (similar to humans) for assessing glucose turnover in NHPs. We have also demonstrated that insulin-resistant, dysmetabolic NHPs respond to the established insulin sensitizer, pioglitazone, thus confirming their use as an ideal pre-clinical translational model to assess insulin sensitizing compounds.

## Introduction

The hyperinsulinemic-euglycemic clamp paradigm [1] is the gold standard for assessing insulin sensitivity. When isotopes are infused in conjunction with an insulin clamp, endogenous glucose production (EGP) and whole body glucose utilization, or rate of disappearance (R_d_) can be measured, allowing for differentiation between sites (hepatic versus peripheral) of insulin resistance or sensitization. While it has been utilized extensively in rodents and dogs, non-human primates remain the closest preclinical species to humans, thus offering the most predictive model for compound efficacy testing as they are thought to develop type 2 diabetes in a very similar manner to that observed in humans [2]. The clamp paradigm (once using isotopes [3]) has been employed in rhesus macaques, although insulin infusion rates and concentrations (400 mU/m^2^ min, ~5000 μU/mL) to assure maximal insulin-stimulated R_d_ and complete suppression of EGP are routinely used [4–9]. Using such pharmacological doses of insulin however, may mask any differentiation of hepatic, and/or perhaps, peripheral insulin sensitivity as well as considerably reduce the therapeutic improvement window for an insulin sensitizing compound.

The thiazolidinedione (TZD), pioglitazone, remains a mainstay of insulin sensitizing therapeutics for the management of type 2 diabetes. Its effects on increasing both hepatic and peripheral insulin sensitivity, as well as hepatic fat content and plasma adiponectin concentrations, in humans are well documented [10–15]. While pioglitazone has been shown to improve glucose clearance in insulin resistant rhesus monkeys during intravenous glucose tolerance tests [16–18], the exact site(s) of action for this improvement were not determined.

First, an appropriate high physiological insulin infusion rate and concentration (similar to those used in human studies) that would elicit appropriate corresponding pharmacodynamic (i.e. glucose infusion rates, GIR) responses was determined. With this insulin infusion rate, the differences in hepatic and peripheral insulin sensitivity between healthy and dysmetabolic (obese and insulin resistant) rhesus monkeys, were documented using stable isotopes, for the first time to our knowledge. Finally, the hypothesis that a short duration of treatment (28 days) of dysmetabolic monkeys with pioglitazone would increase insulin sensitivity and glucose metabolism (and to what extent) was tested.

## Research Design and Methods

### Ethical statement

All animal protocols were approved prior to implementation by the Institutional Animal Care and Use Committee at Merck Research Laboratories (Kenilworth, NJ) and the David H. Murdoch Research Institute (Kannapolis, NC), and all procedures conformed to the requirements of the Animal Welfare Act. Activities related to animal care including housing, feeding, and environmental enrichment were performed in accordance with Institutional Animal Care and Use Committee-approved standard operating procedures at the David H. Murdoch Research Institute (Kannapolis, NC).

### Housing and Husbandry

Animals were housed in rooms with an ambient temperature of 18–26°C, a relative humidity of 40–70% and a 12-hour light-dark cycle. The animals were individually housed in stainless steel wire-bottomed cages with sufficient space (as defined by the Guide for the Care and Use of Laboratory Animals of the Institute for Laboratory Animal Research/National Academies of Science). All animals were fed ad libitum twice a day and were provided with a commercial primate diet. In addition to normal pellet food, fresh fruit was provided daily, and water was freely available at all times. Additional enrichment and welfare were provided; in particular, each animal had at least one enrichment device either suspended on the outside of the cage or an enrichment device on the floor inside of the cage. In addition, each animal was given a variety of food treats and/or chewable vitamins at least three times a week. All animals were also provided with auditory and visual stimulation daily (radio, television or video). All procedures were performed under anesthesia with an intramuscular injection of ketamine hydrochloride (10 mg/kg), on a surgical table under sterile conditions. All efforts were made to minimize suffering. Animals were monitored throughout the study in consultation with the Institution's clinical veterinarian for any signs of change in their physiological and/or psychological state including: changes in responses to people, changes in levels of aggression, changes in quality and consistency of performance during experiments, changes in physical appearance (e.g. body weight and hair coat quality), loss of appetite, and the development of behavioral abnormalities (e.g. self-mutilation and increased frequency of distress calls). No such changes were observed. No animal were sacrificed or euthanized during the course of the described study.

### Subjects

Fifty-five rhesus monkeys (*Macaca mulatta*) of both sexes between 6 and 26 years of age were included in this study. All animals were born in captivity in the United States and were sourced from Merck-approved vendors. The monkeys were individually housed, and consistent care was provided according to the Animal Welfare Act and Animal Welfare Regulations and Guide for the Care and Use of Laboratory Animals of the Institute for Laboratory Animal Research/National Academies of Science, including attention to environmental enrichment. Veterinary care was provided for any animals requiring medical attention. All protocols were approved by the Institutional Animal Care and Use Committees at Merck Research Laboratories (Kenilworth, NJ) and the David H. Murdoch Research Institute (Kannapolis, NC). All animals were fed ad libitum. Based on glucose disappearance rates, fasting glucose and insulin concentrations, and acute insulin responses (AIR) to intravenous glucose tolerance tests (0.25 g/kg), animals were classified as either being healthy (n = 16) or dysmetabolic (n = 39).

### Hyperinsulinemic-euglycemic clamps

All clamp procedures were performed after a 16 hour overnight fast with access to water. Sedation and chemical restraint for the procedure was induced with ketamine hydrochloride (10 mg/kg) and subsequently maintained with a dose of approximately 5–10 mg/kg, provided as needed at 20 to 30 minute intervals. To assess differing pharmacodynamic effects (GIR) of various insulin concentrations, three insulin infusion rate dose-finding studies were conducted in four dysmetabolic monkeys (3 males and 1 female, with ages ranging from 14 to 22 years). A primed continuous infusion of insulin (Humulin; Eli Lilly, Indianapolis, IN) was given for 60 minutes at one rate, and 60 minutes for a second rate. The same four dysmetabolic monkeys underwent three separate, two-step hyperinsulinemic-euglycemic clamps at the following rates: 10 and 20, 40 and 80, or 120 and 400 mU/m^2^ min. All subsequent clamps utilized a primed (350 mU/m^2^ over 10 minutes) continuous insulin infusion rate of 20 mU/m^2^ min. These hyperinsulinemic-euglycemic clamp studies were preceded by a 60 minute primed (6 mg/kg) continuous (0.06 mg/kg min) infusion of [U-^13^C]-D-glucose (99% ^13^C atom percent excess, Sigma Aldrich, St. Louis, MO) to measure glucose turnover, which continued throughout the study. In all studies, plasma glucose was measured every 5 minutes using an Analox glucose analyzer (Analox Instruments, Lunenburg, MA). An intravenous infusion of 20% dextrose was adjusted to maintain the plasma glucose concentration at basal levels (70 mg/dL). Plasma samples were taken every 10 minutes for measurement of ^13^C-glucose enrichment, and every 15–30 minutes during the clamp for measurement of insulin and glycerol.

### Treatment with pioglitazone

30 dysmetabolic monkeys (26 males and 4 females with ages ranging from 10 to 26 years) had hyperinsulinemic-euglycemic clamps performed 3 hours after being dosed with a palatable vehicle (dysmetabolic-baseline). These monkeys were then dosed (in a palatable treat) daily at 7 A.M. for 28 days with pioglitazone (3 mg/kg). The dose of pioglitazone was chosen based on previous results showing that it improved homeostatic model assessment of insulin resistance (HOMA-IR) of dysmetabolic monkeys [16]. Three hours after their final dose on the day 28, they underwent another hyperinsulinemic-euglycemic clamp (dysmetabolic-pioglitazone). Sample size was calculated to detect a 30% change in stead-state glucose infusion rate/steady-state insulin during the clamp, and a 30% change in basal fasting insulin with 80% power. 16 healthy monkeys (15 males and 1 female with ages ranging from 6 to 21 years) had hyperinsulinemic-euglycemic clamps performed 3 hours after being dosed with a palatable vehicle (Healthy).

#### Assays

Plasma insulin, C-peptide, and adiponectin were measured by immunoassay (Meso Scale Discovery, Rockville, MD). Hemoglobin A1c (HbA1c) was measured from whole blood (Siemens Healthcare Diagnostics, Tarrytown, NY). Plasma glycerol (Sigma Aldrich, St. Louis, MO), Glycomark (1,5-anhydroglucitol, GlycoMark, Inc., New York, NY) low density lipoproteins (LDL), high density lipoproteins (HDL) and triglycerides (Carolina Liquid Chemistries Corp., Brea, CA) were measured by an Olympus AU400E chemistry analyzer (Olympus, Center Valley, PA). To measure isotopic enrichment of glucose, glucose was isolated from plasma by mixing 5 μL of plasma and 100 μL of 75% acetonitrile containing 0.1% formic acid. Samples were centrifuged and the supernatant was transferred to a new vial and evaporated to dryness under a heated stream of nitrogen. The dried residue was reacted with 50 μL hydroxylamine-HCl (25 mg per mL of pyridine) at 65°C for 30 min, 50 μL of acetic anhydride was then added and the mixture was heated further at 75°C for 30 min. Following the conversion of glucose to its aldonitrile penta-acetate derivative, the excess reagent was evaporated to dryness and the sample was reconstituted in 100 μL of ethyl acetate [19]. The isotopic enrichment was determined using an Agilent 5973N MS coupled to a 6890 GC oven fitted with an Agilent DB5-MS column (15m x 250μm x 0.15μm). The oven was initially set at 175° C then programmed to increase at 35° C per min to 300°C. Helium carrier flow was set at 1.0 ml x min^-1^ (2 μL of sample is injected using a 5:1 split). Aldonitrile penta-acetate derivative elutes at ~ 1.71 min. The mass spectrometer was set to perform selected ion monitoring of m/z 314 to 319 (10 ms dwell time per ion) in the electron impact ionization mode. Note that although the infused tracer is M+6, the method actually detects the enrichment of the M+5 species. Since there was negligible generation of M+5 via tracer recycling this analytical approach yields a reliable estimate of glucose flux and circumvents the need to apply some other analytical strategy (e.g. chemical ionization [20]).

### Calculations

Clamp R_d_ was measured under steady-state conditions and was calculated using the following equation: R_d_ = *f* x ([IE_infusate_/IE_plasma_]– 1) where *f* is the infusion rate of [U-^13^C]-D-glucose (mg/kg min), and IE is the isotopic enrichment. Basal glucose turnover was calculated with the same equation. Clamp EGP was calculated by subtracting clamp GIR from R_d_. HOMA-IR was calculated using the following equation: HOMA-IR = (fasting glucose x fasting insulin)/22.5, where glucose is in mM and insulin is in μU/mL. Insulin clearance was calculated by dividing the insulin infusion rate (mU/m^2^ min) by plasma concentration (mU/mL).

### Statistical Analysis

To assess the effect of pioglitazone, chronic measurements were compared with baseline values for dysmetabolic monkeys using a mixed effects model with period as a fixed effect and animal ID as a random effect. Vehicle induced responses from healthy and dysmetabolic monkeys at baseline were compared using linear regression. All responses except Clamp EGP, EGP % Suppression, steady-state (ss) EGP/ss-insulin, and change in glycerol during clamp procedure (steady state level—basal level) were natural log-transformed to satisfy distributional assumptions. All results are presented as mean +/- SEM. Effects with one sided p value <0.05 were considered statistically significant. Statistical calculations were performed with SAS for Windows (version 9.3, SAS Institute, Cary, NC).

## Results

### Insulin infusion rate dose finding studies

**Fig 1:**
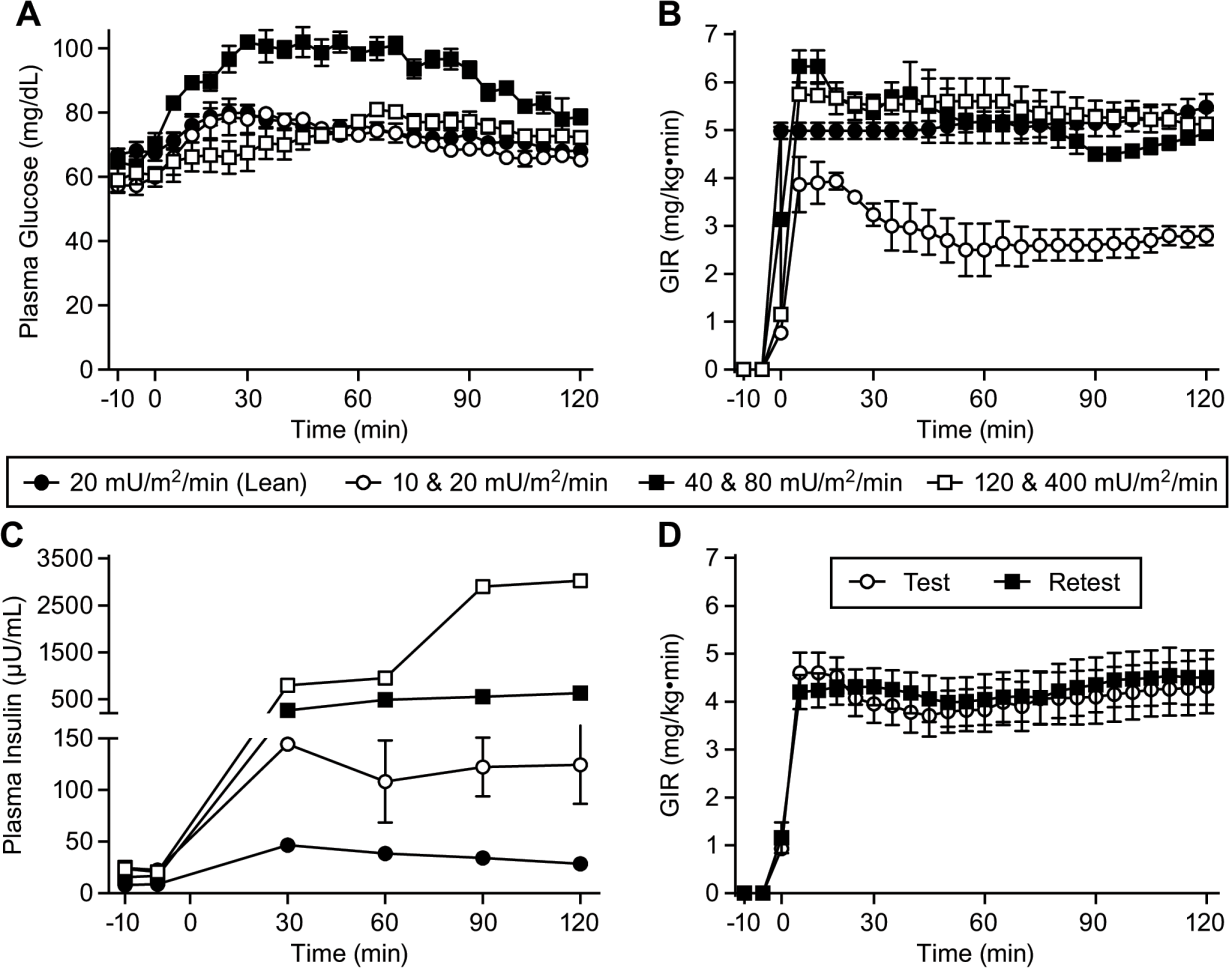
Insulin infusion rate dose-finding and reproducibility studies. Time courses of (A) plasma glucose, (B) glucose infusion rate, and (C) plasma insulin concentrations during two hour (one hour per insulin infusion rate) hyperinsulinemic-euglycemic clamps. (D) Reproducibility of glucose infusion rate requirements during two separate hyperinsulinemic-euglycemic clamps (both at 20 mU/m^2^ min) separated by two weeks. Values are means ± SEM.

In attempts to find an insulin infusion rate that would not elicit maximal pharmacodynamic (GIR) effects and offer a window for improvement upon in dysmetabolic monkeys, several insulin infusion rates (10, 20, 40, 80, 120, and 400 mU/m^2^ min for only 1 hour) were tested in 4 dysmetabolic monkeys to compare the GIR requirements to that of healthy monkeys. Glucose was clamped at ~70 mg/dL in all groups (Fig 1A). By the end of the 1 hour insulin infusion periods, the 10, 20, 40, 80, 120, and 400 mU/m^2^ min infusions resulted in plasma insulin concentrations of 108±40, 124±43, 262±83, 631±41, 795±78, and 3027±77 μU/mL, respectively (Fig 1C). By the end of the 1 hour insulin infusion periods, the 10, 20, 40, 80, 120, and 400 mU/m^2^ min infusions required a GIR of 2.5±0.6, 2.8±0.2, 5.2±0.4, 4.9±0.1, 5.6±0.5, and 5.1±0.3 mg/kg min, respectively (Fig 1B). Since it appeared that a maximal pharmacodynamic effect was being achieved between 20 and 40 mU/m^2^ min, we chose to test the effects of 20 mU/m^2^ min in 16 healthy monkeys in a 2 hour hyperinsulinemic-euglycemic clamp. As is shown in Fig 1B, a maximal effect (5.5 mg/kg min) was achieved in the healthy monkeys, while in dysmetabolic monkeys, the effect (albeit with only 1 hour of the infusion) was reduced by ~50% (2.8 mg/kg min), offering a significant window for improvement. Interestingly, the insulin levels achieved in the healthy monkeys were significantly lower than the dysmetabolic monkeys, suggesting a difference in insulin clearance. We next tested the reproducibility of this pharmacodynamic effect of 20 mU/m^2^ min by clamping 10 dysmetabolic monkeys (6 males and 4 females with ages ranging from 15 to 22 years), two separate times (with 2 weeks rest in between). As shown in Fig 1D, the GIR results were reproducible.

### Body weight, glucose, insulin, and HOMA-IR

**Fig 2:**
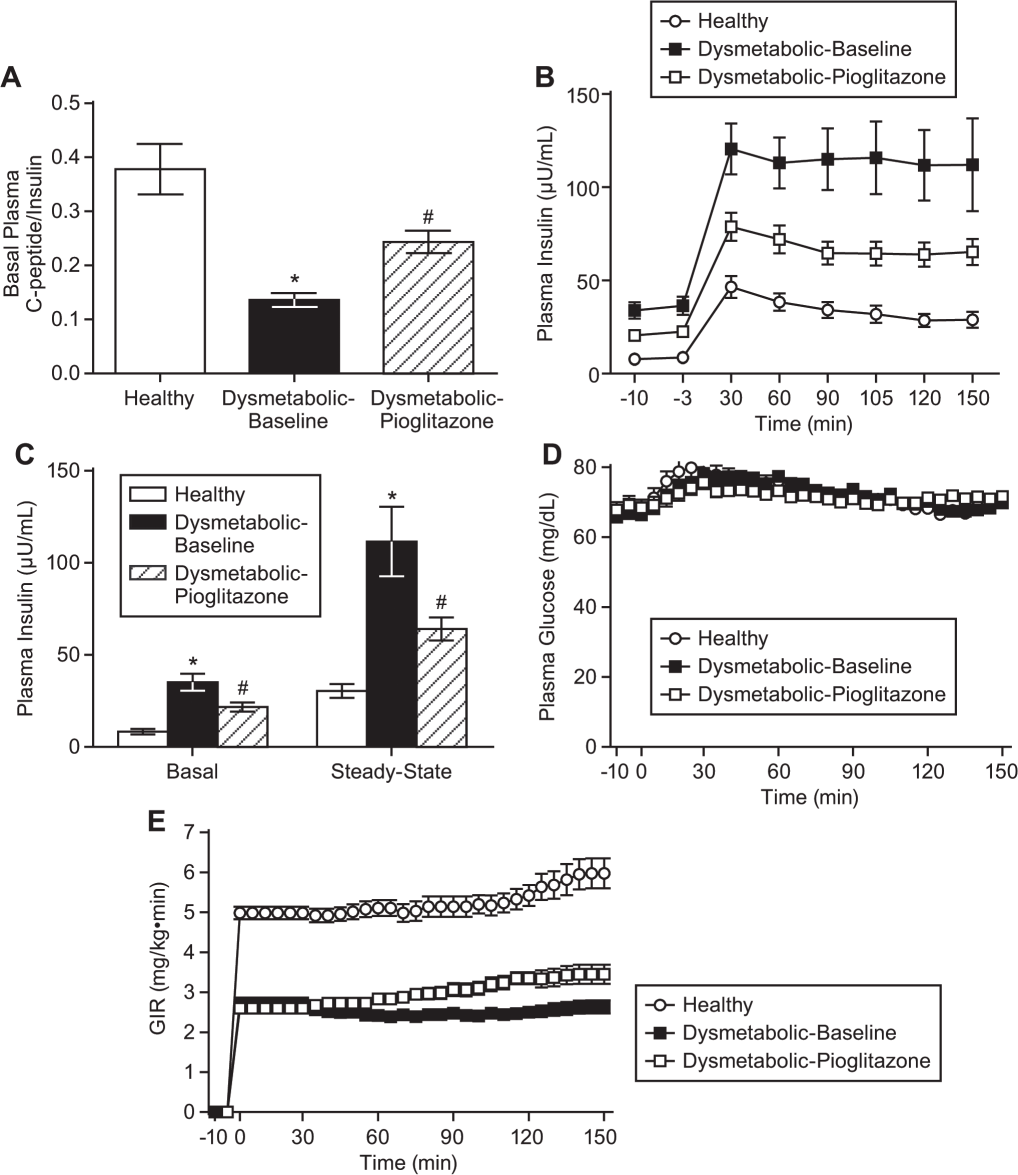
Insulin sensitivity measures of healthy, dysmetabolic, and dysmetabolic monkeys treated with pioglitazone for 28 days. (A) Basal plasma C-peptide/insulin ratio. (B) Time course of insulin, (C) period insulin, and time courses of (D) plasma glucose and (E) glucose infusion rates during hyperinsulinemic-euglycemic clamps. Values are means ± SEM. *p<0.05 versus healthy; #p<0.05 versus dysmetabolic-baseline

Healthy monkeys weighed significantly less than dysmetabolic, and 28 days of pioglitazone treatment (3 mg/kg day) had no effect on body weight (body weights were 11.2±0.5, 16.6±0.5, and 16.3±0.4 kg, respectively, p<0.05 between healthy and dysmetabolic groups). Monkeys were >99% compliant (data not shown) for pioglitazone consumption which resulted in concentrations of 2.713±0.415 and 0.531±0.060 μM at 3 hours (peak) and 20 hours (trough) post-dosing, respectively. The ratio of basal C-peptide/insulin (considered to be a marker for hepatic insulin clearance) was significantly increased in dysmetabolic monkeys, and pioglitazone treatment significantly decreased this (Fig 2A). Basal insulin concentrations were significantly increased in dysmetabolic monkeys, and pioglitazone treatment significantly decreased this. As a result, insulin levels significantly differed between groups during the clamp (Fig 2B and 2C). There were no significant differences in basal or clamp glucose concentrations (Fig 2D). Experiments were well executed, with glucose being clamped at 70 mg/dL in all groups with a steady GIR (Fig 2E). As expected, healthy monkeys were significantly more insulin sensitive than dysmetabolic monkeys, and pioglitazone improved insulin sensitivity as measured by HOMA-IR (values were 1.5±0.3, 5.8±0.9, and 3.7±0.5, respectively, p<0.05 between all groups).

### Glucose turnover and insulin clearance

**Fig 3:**
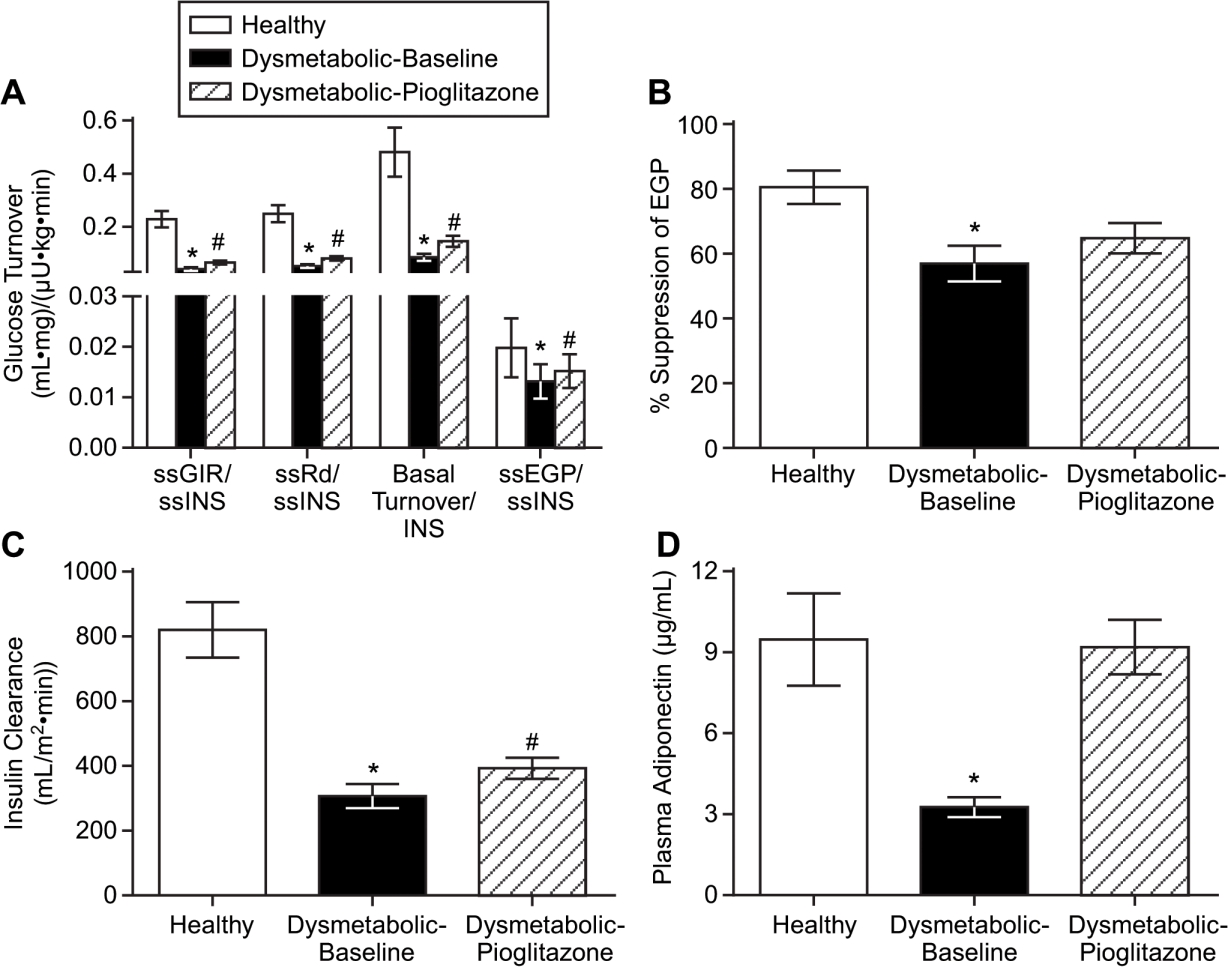
Glucose turnover data in healthy, dysmetabolic, and dysmetabolic monkeys treated with pioglitazone for 28 days. (A) Steady-state glucose fluxes normalized for steady-state insulin concentrations, (B) percent suppression of EGP, and (C) insulin clearance during hyperinsulinemic-euglycemic clamps. (D) Fasting adiponectin concentrations. Values are means ± SEM. *p<0.05 versus healthy; #p<0.05 versus dysmetabolic-baseline

Steady-state (ss) R_d_ was 6.0±0.2, 3.4±0.1, and 4.1±0.2 mg/kg min, while ss-EGP was 0.5±0.1, 0.8±0.1, and 0.7±0.1 mg/kg min in Healthy, dysmetabolic-baseline, and dysmetabolic-pioglitazone, respectively (data not shown). Because basal and clamp insulin levels were different between all groups, we calculated insulin clearance during the clamp, and found that it was significantly decreased in the dysmetabolic compared to healthy monkeys (Fig 3C). Pioglitazone moderately, yet significantly, increased it. Because of the difference in ss-clearance (and hence ss-insulin levels), glucose turnover data were normalized to ss-insulin concentrations. Healthy monkeys had a significantly higher basal glucose turnover, indicative of higher basal glucose utilization compared to dysmetabolic monkeys, and pioglitazone increased it in dysmetabolic monkeys (Fig 3A). The ss-GIR/ss-insulin required to maintain the glucose clamp was significantly decreased in dysmetabolic versus healthy monkeys, and pioglitazone increased it as well. The same differences were evident for ss-Rd/ss-insulin and ss-EGP/ss-insulin, although pioglitazone failed to significantly increase the % suppression of EGP during the clamp (Fig 3B).

### Other plasma measurements

The insulin sensitizing adipokine, adiponectin, was significantly decreased in dysmetabolic versus healthy monkeys, and pioglitazone completely normalized plasma values (Fig 3D). Plasma triglycerides, HDL, LDL, GlycoMark (reflects 1 to 2 week glycemic control), and HbA1c were all significantly different comparing dysmetabolic versus healthy monkeys. Pioglitazone significantly improved triglycerides, HDL, GlycoMark, and HbA1c (Table 1).

**Table 1:** Plasma lipids and glycemic control markers.

	Triglycerides (mg/dL)	HDL (mg/dL)	LDL (mg/dL)	Glycomark (μg/mL)	HbA1c (%)
Healthy	26±3	92±6	46±3	1.80±0.21	4.42±0.07
Dysmetabolic-Baseline	109±9*	64±3*	60±5*	1.23±0.12*	5.06±0.16*
Dysmetabolic-Pioglitazone	79±14†	72±3†	55±4	1.57±0.13†	4.77±0.13†

Values are means ± SE.

*p<0.05 versus Healthy;

†p<0.05 versus Dysmetabolic-Baseline

## Discussion

In this study, we have determined an insulin infusion rate that results in high physiological insulin concentrations (~120 μU/mL) in dysmetabolic monkeys (similar to concentrations seen in human clamp studies [21, 22]). At this concentration, a pharmacodynamics response (GIR) was observed, which is ~50% less than that of healthy monkeys, providing a substantial window for improvement with a pharmaceutical intervention. This 50% decrement in the pharmacodynamic response to insulin in dysmetabolic versus healthy monkeys was further dissected into quantitative differences in hepatic (EGP) and peripheral (R_d_) insulin sensitivity using stable isotopes. These differences in both hepatic and peripheral insulin sensitivity found with this insulin infusion rate are of particular importance, because it not only allows for a window for improvement in peripheral, but also hepatic insulin sensitivity. This was something not possible with previous pharmacological infusion rates used in hyperinsulinemic-euglycemic clamps with rhesus monkeys [4–9]. Finally, we confirmed that this is a suitable insulin infusion rate for determining therapeutic efficacy by documenting significant improvements on both hepatic and peripheral insulin sensitivity using the established insulin sensitizer, pioglitazone.

The degree of hepatic and peripheral insulin resistance in patients with type 2 diabetes [23–29] or impaired glucose tolerance (IGT, [21, 30]) has been well documented with the use of tracer dilution techniques. The ability of insulin to stimulate R_d_ is diminished by ~40% in patients with IGT [21, 22, 30]; while in type 2 diabetic patients it is diminished by ~40–50% [24, 25, 28, 29] in the presence of similar hyperinsulinemic levels achieved in our studies. Interestingly, the difference in insulin-stimulated R_d_ between healthy and dysmetabolic monkeys is also diminished by 43% (6 versus 3 mg/kg min), remarkably similar to that reported in insulin resistant patients. However, it must be noted that there were large differences in steady-state insulin concentrations in our clamps, which were not present in the human studies. When taking that into account (normalizing ss-R_d_ for ss-insulin), the difference is even larger in the dysmetabolic monkeys (80%, 0.249 versus 0.052 [mL mg]/[μU kg min]).

It has been shown previously that pioglitazone enhances both hepatic and peripheral insulin sensitivity in both type 2 diabetic patients [10, 11, 13, 15] and patients with IGT [31, 32]. Four months of daily pioglitazone treatment (30 mg) increased insulin-stimulated R_d_ (at similar steady-state insulin concentrations to this study) by 14% in IGT patients [32], while six months of daily pioglitazone treatment (45 mg) increased it by 28% in IGT patients with polycystic ovary syndrome [31]. Several studies using two step hyperinsulinemic-euglycemic clamps (steady-state insulin concentrations of half to 3-fold those seen in this study) in type 2 diabetic patients have shown daily treatment with pioglitazone (45 mg) for 4 months increased insulin-stimulated R_d_ by 17–44%, while also enhancing insulin-mediated suppression of EGP by [10, 11, 13, 15]. Similar to these findings in patients with IGT or type 2 diabetes, we show that shorter term (28 days) treatment of dysmetabolic monkeys with pioglitazone (3 mg kg^-1^ day^-1^) increases insulin-stimulated R_d_ by 62% and enhanced insulin-mediated suppression of EGP by 15%.

In mice, adiponectin has been shown to increase muscle fat oxidation (reducing triglyceride content), improve muscle insulin sensitivity, and decrease basal EGP [33–36]. Circulating adiponectin concentrations are strongly inversely correlated with insulin resistance and risk for type 2 diabetes [37, 38] even though a direct causal effect remains uncertain [39]. As has previously been shown [5], a decrease in plasma adiponectin accompanied the decrease in insulin sensitivity of dysmetabolic rhesus monkeys, mirroring the changes seen in insulin resistant patients associated with hepatic [12, 14] or visceral [11] fat content and insulin resistance [13]. Also similar to findings from these studies of type 2 diabetic patients, or IGT patients [31] treated with pioglitazone, dysmetabolic rhesus monkeys treated with pioglitazone for 28 days not only significantly increased both hepatic and peripheral insulin sensitivity, but also completely normalized circulating adiponectin concentrations to that of healthy monkeys (Fig 3D). One proposed mechanism to explain the metabolic effects of adiponectin is to enhance expression of peroxisome proliferator activator receptor-α, leading to increased fat oxidation [36]. It is interesting to speculate that pioglitazone decreases hepatic and visceral fat in dysmetabolic monkeys like it does in type 2 diabetic patients to increase hepatic insulin sensitivity. Unfortunately without biopsies or magnetic resonance spectroscopy data, this remains only a hypothesis. It must also be noted that it has previously been shown that while there is no defect in insulin’s regulation of hepatic glycogen metabolism in dysmetabolic monkeys [6], there is a defect in insulin-stimulated protein kinase B (PKB/Akt) activity [8], perhaps suggesting that hepatic insulin resistance in them may manifest in the regulation of gluconeogenesis via PKB and forkhead box protein O1[40]. These dysmetabolic monkeys did not have overt type 2 diabetes. Thus, we have documented that hepatic insulin resistance as a whole (in comparison to hepatic insulin signaling) appears to not be as late of an event in the progression to overt diabetes as previously suggested using pharmacological insulin doses [3, 6, 8], although the more severe peripheral insulin resistance in these monkeys agrees with the notion that it does develop first [41].

In accordance with peripheral (muscle) insulin resistance developing first, and therefore having a larger deficit (as measured by the hyperinsulinemic-euglycemic clamp), insulin-stimulated activation of skeletal muscle atypical protein kinase Cs-(ζ/λ/ι), insulin receptor substrate (IRS)-1, phosphatidylinositol (PI) 3-kinase, PKB, and glycogen synthase have all been shown to be decreased in dysmetabolic monkeys [8, 41, 42]. Not surprisingly, given TZDs’ known effect to increase muscle insulin sensitivity, treatment of dysmetabolic monkeys with various TZDs has been shown to enhance insulin-stimulated atypical protein kinase C[8] and glycogen synthase activities [43], while rosiglitazone has also been shown to increase 5’-AMP-acitivated protein kinase activity as well [8]. Taken together, pioglitazone’s increase of insulin-stimulated R_d_ can be explained by any of these (or a combination of) enhancements to muscle insulin signaling that have been shown previously in dysmetabolic monkeys treated with TZDs.

The insulin resistance in these dysmetabolic monkeys was clearly evidenced by the fasting hyperinsulinemia, as opposed to hypoinsulinemia resulting from β–cell exhaustion in overt type 2 diabetic monkeys [3]. Numerous investigators have found that as much as 75% of the hyperinsulinemia in obese, insulin resistant patients, appeared to be the result of decreased hepatic insulin clearance [44–47], a phenomenon also noted in dysmetabolic monkeys [48]. Under fasting conditions, hepatic insulin clearance can be calculated by the ratio of fasting C-peptide to insulin in peripheral blood [49]. As previously demonstrated in monkeys, a similar decrease in fasting C-peptide/insulin that precedes overt type 2 diabetes [48] was seen in our dysmetabolic monkeys, and pioglitazone treatment significant increased this ratio (Fig 2A). Further evidence of decreased hepatic insulin clearance was seen during the clamp (Fig 3C), again with pioglitazone treatment significantly improving the decrease insulin clearance seen in dysmetabolic monkeys. This again may be indicative of the well documented pioglitazone-induced decreases in hepatic fat content in type 2 diabetic patients [11, 12, 14], and its associated changes in insulin clearance [50–52].

As previously documented in type 2 diabetic patients [10–15, 32], pioglitazone also significantly decreased the elevated plasma triglycerides and HbA1c in these dysmetabolic monkeys. In contrast to what was seen in these patients though, was that pioglitazone also significantly elevated HDL of dysmetabolic monkeys. These findings are similar to those previously reported for pioglitazone [16] or dual peroxisome proliferator activator receptor- α/γ agonists [9, 53] in dysmetabolic monkeys.

In this study we have determined non-pharmacological insulin infusion rates that elicit a maximal pharmacodynamic response (GIR) in normal monkeys, but only half of that response in insulin resistant dysmetabolic monkeys, offering a large window for improvement with pharmaceutical intervention. Using stable isotopes, we have quantified the degree of both hepatic (EGP) and whole body (R_d_) insulin resistance in dysmetabolic versus healthy monkeys for the first time. Finally, we have documented the improvement in hepatic and peripheral insulin sensitivity in dysmetabolic monkeys treated short term (28 days) with the established insulin sensitizer, pioglitazone. These studies confirm the suitability of dysmetabolic rhesus monkeys as an ideal translational preclinical animal model for the assessment of insulin sensitizing compounds.

## Supporting Information

S1 Checklist Completed ARRIVE (Animal Research: Reporting of In Vivo Experiments) guidelines describing laboratory-based animal research. (PDF)
